# A quantitative method to assess extrasynaptic NMDA receptor function in the protective effect of synaptic activity against neurotoxicity

**DOI:** 10.1186/1471-2202-9-11

**Published:** 2008-01-24

**Authors:** C Peter Bengtson, Oliver Dick, Hilmar Bading

**Affiliations:** 1 Department of Neurobiology, Interdisciplinary Centre for Neurosciences (IZN), University of Heidelberg, 69120 Heidelberg, Germany

## Abstract

**Background:**

Extrasynaptic NMDA receptors couple to a CREB shut-off pathway and cause cell death, whereas synaptic NMDA receptors and nuclear calcium signaling promote CREB-mediated transcription and neuronal survival. The distribution of NMDA receptors (synaptic versus extrasynaptic) may be an important parameter that determines the susceptibility of neurons to toxic insults. Changes in receptor surface expression towards more extrasynaptic NMDA receptors may lead to neurodegeneration, whereas a reduction of extrasynaptic NMDA receptors may render neurons more resistant to death. A quantitative assessment of extrasynaptic NMDA receptors in individual neurons is needed in order to investigate the role of NMDA receptor distribution in neuronal survival and death.

**Results:**

Here we refined and verified a protocol previously used to isolate the effects of extrasynaptic NMDA receptors using the NMDA receptor open channel blocker, MK-801. Using this method we investigated the possibility that the known neuroprotective shield built up in hippocampal neurons after a period of action potential bursting and stimulation of synaptic NMDA receptors is due to signal-induced trafficking of extrasynaptic NMDA receptors or a reduction in extrasynaptic NMDA receptor function. We found that extrasynaptic NMDA receptor-mediated calcium responses and whole cell currents recorded under voltage clamp were surprisingly invariable and did not change even after prolonged (16 to 24 hours) periods of bursting and synaptic NMDA receptor activation. Averaging a large number of calcium imaging traces yielded a small (6%) reduction of extrasynaptic NMDA receptor-mediated responses in hippocampal neurons that were pretreated with prolonged bursting.

**Conclusion:**

The slight reduction in extrasynaptic NMDA receptor function following action potential bursting and synaptic NMDA receptor stimulation could contribute to but is unlikely to fully account for activity-dependent neuroprotection. Other factors, in particular calcium signaling to the nucleus and the induction of survival promoting genes are more likely to mediate acquired neuroprotection.

## Background

Synaptic and extrasynaptic NMDA receptors are, respectively, coupled to survival and cell death pathways, which involves their opposing effects on the cAMP response element binding protein (CREB) [1-6] and their regulation of overlapping but distinct genomic programs recently revealed by a whole genome transcriptome analysis [7]. The differential role of NMDA receptors provides a new concept explaining how the same receptor, dependent on its location, can couple to both survival and death. This concept represents an alternative to the "Ca^2+ ^load" hypothesis, which attempts to assign a toxic threshold to Ca^2+ ^influx associated with NMDA receptor activation [8,9]. Precisely how NMDA receptors differentially regulate the activity of CREB or signaling molecules such as the extracellular signal-regulated kinases 1 and 2 (ERK1/2) is unknown, but differences in the NMDA receptor subunit composition and/or differences in signaling complexes associated with synaptic versus extrasynaptic NMDA receptors may be important [5,10-13].

The toxic effects of extrasynaptic NMDA receptor activation can be counteracted to some extent by prior activation of synaptic NMDA receptors. For example, prolonged periods action potential (AP) bursting-induced with the GABA_A _receptor antagonist, bicuculline in cultured hippocampal networks robustly activates synaptic NMDA receptors, which protects against subsequent NMDA-induced excitotoxicity [14] as well as against pro-apoptotic stimuli such as serum deprivation [6] or staurosporine treatment [4]. Similarly, minor ischemic events or preconditioning systemic doses of NMDA are neuroprotective [15-19]. The neuroprotective effects of preconditioning neurons with low concentrations of NMDA are mediated, at least in cultured hippocampal networks, via AP-induced stimulation of synaptic NMDA receptors [20]. The molecules responsible for synaptic NMDA receptor-induced survival represent potential clinical targets to reduce neuron loss associated with pathological conditions including stroke and neurodegenerative diseases in which NMDA receptor-mediated excitotoxicity has been implicated [21-27].

NMDA receptor-mediated neuroprotection appears to involve multiple players including nuclear Ca^2+ ^signaling, CREB, nuclear factor kappa B, ERK1/2, Akt1, phosphatidylinositol 3-kinase, protein kinase C epsilon, and brain-derived neurotrophic factor [6,15-17,19,28,29]. Given the central role of extrasynaptic NMDA receptors in cell death, it is also conceivable that signal-induced changes in surface expression or function of this pool of receptors could profoundly affect the susceptibility of neurons to toxic insults. The surface expression of NMDA receptors (presumably both synaptic and extrasynaptic receptors) is dynamic, whereby receptor endocytosis, exocytosis, and lateral movement are strongly regulated by activity [30-33]. The first step in determining whether changes in the relative distribution of NMDA receptors (synaptic versus extrasynaptic) are associated with and responsible for activity and NMDA receptor-induced neuronal survival, requires a method that allows the precise quantitative assessment of extrasynaptic NMDA receptor function in individual neurons.

Techniques for the identification of the extrasynaptic NMDA receptor pool in brain slices are emerging [34,35]. However, considerable advances have been made in isolating extrasynaptic NMDA receptor function in cultured neurons. Such studies have employed a protocol, which specifically blocks synaptic NMDA receptors with MK-801. MK-801 is a use-dependent open channel NMDA receptor blocker, which enters the channel only after its activation but then becomes trapped inside the pore to "irreversibly" block the receptor as long as the receptor is not re-activated to release the blocker [36,37]. Extrasynaptic NMDA receptor-mediated currents have been measured in single neurons isolated in micro-island cultures after blocking autaptic synapses with MK-801 during synaptic stimulation [38]. Techniques for quantifying extrasynaptic NMDA receptor-mediated currents in cultures of neuronal networks have also been developed but the parameters necessary for use-dependent blockade of NMDA receptors require refinement. Mass activation of synaptic NMDA receptors in neuronal networks of hippocampal cultures can be achieved using the GABA_A _receptor antagonist, bicuculline, which initiates recurrent synchronous bursting [4,5,39]. MK-801 application during bicuculline-induced bursting in hippocampal cultures provides a use-dependent blockade of synaptic NMDA receptors allowing the extrasynaptic NMDA receptor population to be subsequently activated with bath applied NMDA [13,40].

The aim of this study was to investigate the possibility that the known neuroprotection afforded by synaptic NMDA receptor activation is mediated by changes in the surface expression and/or function of extrasynaptically localized NMDA receptors. A method based on previously used protocols was refined and verified in order to isolate extrasynaptic NMDA receptors and quantify their function in individual neurons that are part of a complex neuronal network. We found a surprisingly small neuron-to-neuron variation in extrasynaptic NMDA receptor function. Overnight network bursting, stimulating synaptic NMDA receptors, led to a measurable but very small loss of extrasynaptic NMDA receptor function, which seemed dwarfed by the dramatic neuroprotective effect of this treatment.

## Results

### MK-801 treatment in bursting networks effectively isolates the extrasynaptic pool of NMDA receptors

**Figure 1:**
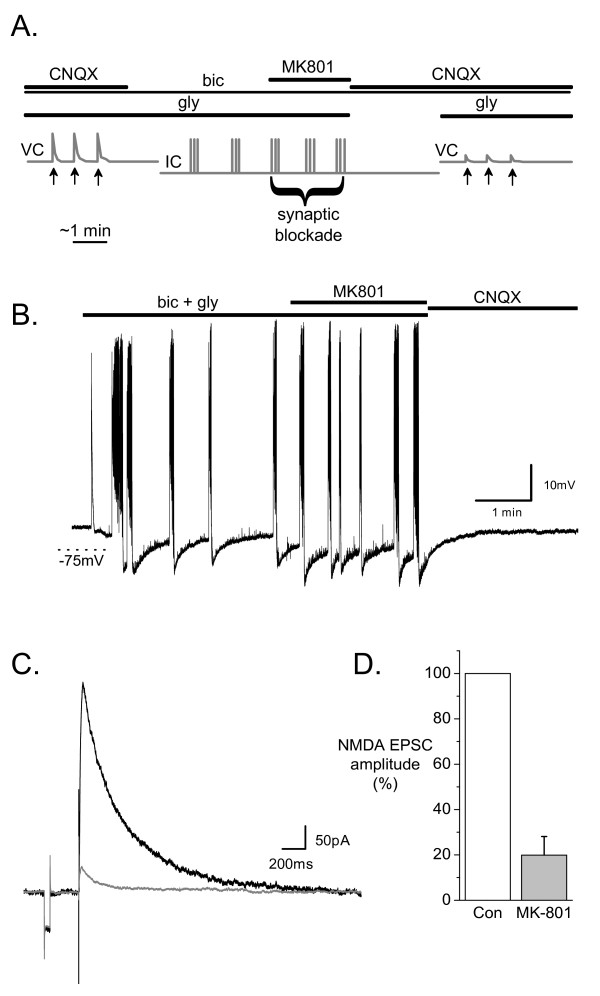
**MK-801 effectively blocks synaptic NMDA receptors during bicuculline induced bursting**. (A) The MK-801 protocol used to selectively block synaptic NMDA receptors is shown schematically (see Methods). NMDA receptor-mediated EPSCs were evoked by single stimuli (arrows) and measured in voltage clamp mode (VC) at +40 mV in the presence of 10 μM CNQX, 50 μM bicuculline (bic) and 10 μM glycine (gly). Following CNQX washout, bursting returned as was recorded in current clamp mode (IC) before 10 μM MK801 was applied for 4 to 8 bursts (42 to 188 spikes, 2.2 to 6.2 min) to selectively block synaptic NMDA receptors (synaptic blockade). Bursting was then silenced in CNQX in the absence of glycine to prevent unblocking of NMDA receptors. EPSCs were recorded under the same stimulation parameters. (B) Representative recording from the IC component of the protocol. (C) NMDA receptor-mediated EPSCs recorded before (black trace) and after (grey trace) the MK801 protocol recording shown in B. (D) Cumulative data from 5 neurons showing the EPSC amplitude before (control) and after the MK-801 protocol (MK-801). Bars represent the mean and whiskers the SEM.

To establish the necessary parameters to isolate extrasynaptic NMDA receptors in hippocampal cultures, we first examined the blockade of synaptic NMDA receptor evoked EPSCs by MK-801 during bicuculline-induced bursts of APs. The protocol is described in the methods section and schematically represented in Figure 1A. Both before and after the MK-801 blocking protocol EPSCs were measured at +40 mV in the presence of 1 mM Mg^2+ ^as well as GABA_A _and AMPA receptor antagonists. These recordings were not feasible at negative holding potentials in the absence of Mg^2+ ^due to the appearance of multiple NMDA receptor-mediated EPSCs evoked by single stimuli (data not shown). Although NMDA and AMPA components of EPSCs can be differentiated from each other, the continuous barrage of EPSCs in AP bursting cultures necessitated the blockade of AMPA receptors. CNQX always blocked all burst activity (Figure 1B) and was applied during the MK-801 washout period to ensure that synaptic NMDA receptors remained blocked. Under these conditions, MK-801 (10 μM) application for 2 to 6 min encompassing 4 to 8 bursts was sufficient to reduce NMDA receptor-mediated evoked EPSCs to 19.9 ± 8.2% of their control amplitude (n = 5, Figure 1C,D). Although such evoked EPSCs probably do not reflect the response of all NMDA receptor containing synapses in each cell, they represent a random subset of such synapses, most of which were blocked by brief MK-801 treatment during bicuculline induced bursting.

**Figure 2:**
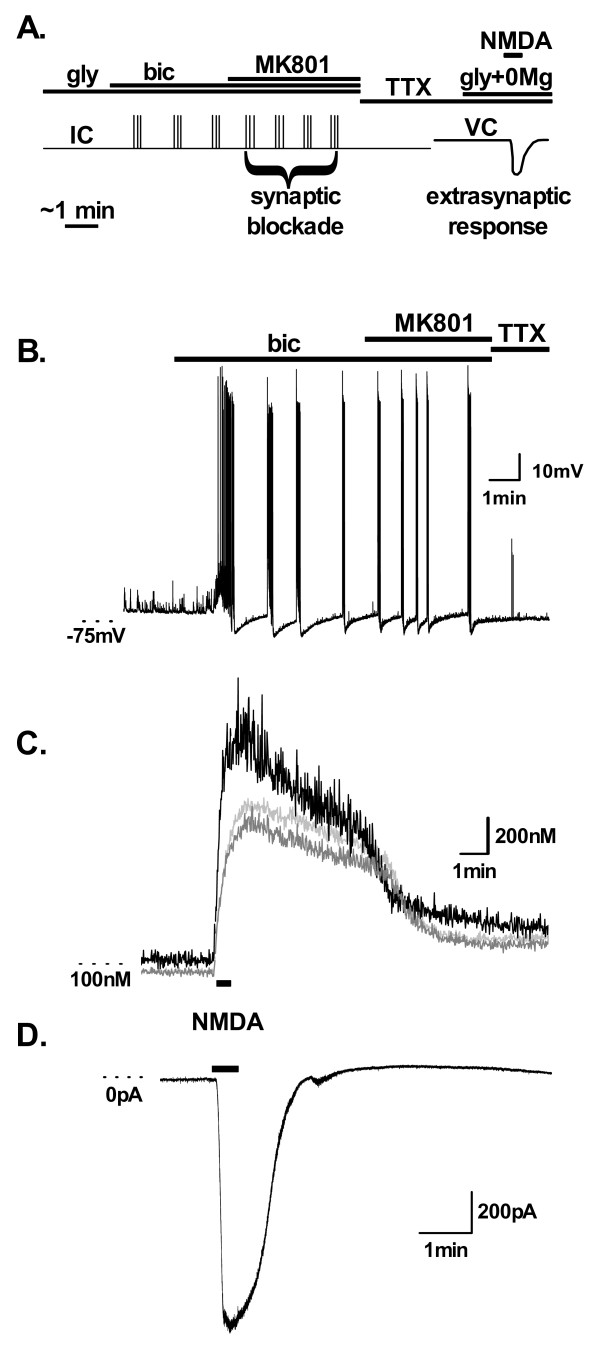
**Ca^2+ ^and current responses of the total extrasynaptic NMDA receptor pool in single hippocampal neurons**. (A) The protocol used to isolate and measure extrasynaptic NMDA receptor responses is shown schematically. Application of 50 μM bicuculline in the presence of 10 μM glycine initiates recurrent AP bursting as recorded in current clamp (IC). 10 μM MK-801 is then applied for 4 to 20 bursts (48 to 163 action potentials, 2 to 10 min) to block synaptic NMDA receptors (synaptic blockade) before halting synaptic activity with TTX in the absence of glycine. In voltage clamp (VC, Vhold = -71 mV) the extrasynaptic pool of receptors is then activated with a 30 s application of 100 μM NMDA in the absence of Mg^2+ ^and the presence of 10 μM glycine. Spontaneous activity of all cells was measured in cell attached mode before commencing experiments. (B-D) Example recordings from the same cell are shown for the (B) IC and (C, D) VC components of the recording. Ca^2+ ^recordings in C show the isolated extrasynaptic NMDA receptor response measured in the nucleus (light grey), soma (grey) and a dendritic region 50 μm from the soma (black) measured with cell impermeant bis-FURA2.

Having established the parameters necessary to selectively block synaptic NMDA receptors, we utilized this protocol to functionally quantify the entire extrasynaptic NMDA receptor pool in single neurons using simultaneous voltage clamp electrophysiology and fluorescent Ca^2+ ^imaging. Synaptic NMDA receptors were blocked by MK-801 during bicuculline induced AP bursting in current clamp mode (Figure 2A,B). MK-801 was washed out of the bath in the absence of glycine as in the EPSC protocol however CNQX was replaced with TTX to block all AP-induced neurotransmitter release in pre-synaptic cells. Current and Ca^2+ ^responses to a 30 s rapid bath perfusion (6 ml/min) of 100 μM NMDA were then measured at a holding potential of -71 mV (including a -11 mV correction for junction potential) in 10 μM glycine and in the absence of Mg^2+ ^(n = 19, Figure 2C,D). The extrasynaptic NMDA receptor pool was measured in this manner in one cell per coverslip.

### Protective effects of AP bursting against NMDA toxicity

**Figure 3:**
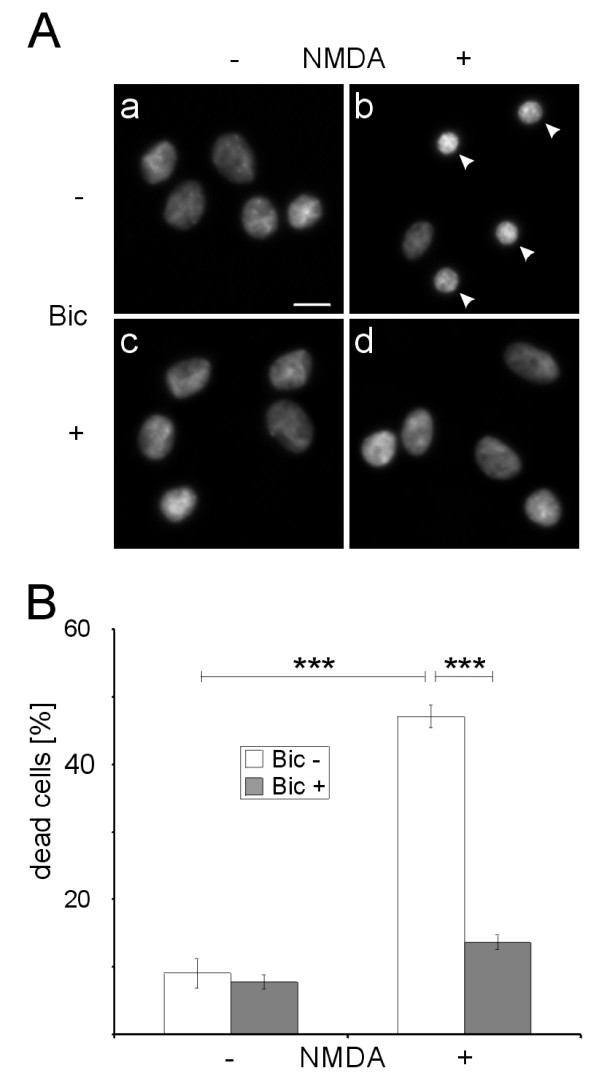
**AP bursting protects against NMDA-induced cell death**. (A) Hippocampal neurons with (+) or without (-) 16 h bicuculline pretreatment (Bic) were incubated with (+) or without (-) 20 μM NMDA for 10 min. Five hours after NMDA washout the cells were fixed and stained with Hoechst 33258. Arrowheads indicate dead cells. Scale bar is equal to 10 μm. (B) Evaluation of neuronal cultures following treatment with bicuculline and NMDA showed the protective effect of bicuculline pretreatment on NMDA induced cell death. The graph represents the mean and SEM of five independent experiments. *** p < 0.001 independent samples t-test.

As a first step towards analyzing a possible mechanistic link between extrasynaptic NMDA receptor function and acquired neuroprotection, we established conditions that promote neuronal survival. Hippocampal neurons were treated overnight with bicuculline, which induced recurrent bursts of APs that are associated with synaptic NMDA receptor-dependent Ca^2+^-transients throughout the neuron [3]. Neurons were subsequently challenged with NMDA (20 μM) for 10 min, which triggers excitotoxic cell death. In untreated cultures, 9 ± 2% of cells showed condensed nuclei indicative of cell death; NMDA treatment increased this cell death to 47 ± 2%. In cultures that were pre-treated with AP bursting for 16 h before NMDA application, only 14 ± 1% of the neurons showed condensed nuclei and were counted as dead. AP bursting for 16 h without subsequent NMDA treatment had no significant effect on cell death (8 ± 1% of cells were dead) (all experiments at 37°C, 3 coverslips per group repeated in 5 independent experiments, Figure 3). These results demonstrate that recurrent network AP bursting protects neurons from subsequent NMDA-induced cell death.

### Ca^2+ ^responses to toxic NMDA treatment

**Figure 4:**
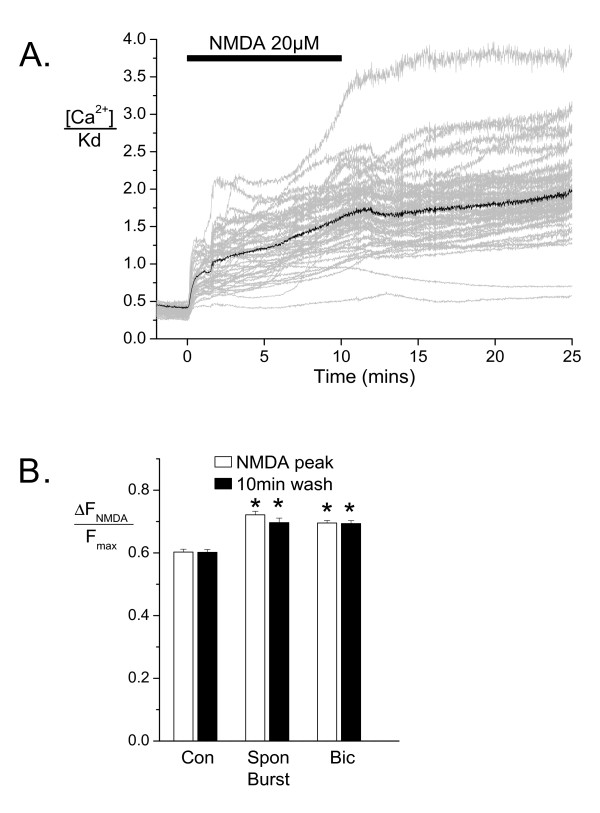
**Imaging of Ca^2+ ^responses to excitotoxic NMDA receptor activation**. (A) Shown are representative Ca^2+ ^responses to 20 μM NMDA at 37°C from a representative overnight vehicle treated culture. Grey traces show the results recorded simultaneously from the soma (including nucleus) of multiple cells in the same field of view and black traces show the average of these cells. (B) Cumulative data measured at the peak of the response during NMDA application and at a point 10 min after washout of NMDA are shown from vehicle treated (Con, n = 250 cells on 5 coverslips) spontaneously bursting (Spon Burst, n = 128 cells on 2 coverslips) and bicuculline-treated coverslips (Bic, n = 316 cells on 6 coverslips). Peak NMDA responses and Ca^2+ ^levels 10 min after NMDA washout in the bicuculline and spontaneously bursting groups were larger than those of the control groups (p < 0.01, ANOVA, Tukey's posthoc tests).

Since Ca^2+ ^overload has been suggested as a trigger for cell death in ischemia and NMDA receptor activation [8,41,42], we measured the Ca^2+ ^load at the soma activated by this toxic 10 min treatment with 20 μM NMDA at 37°C (Figure 4A) and compared it with hippocampal neurons that had been treated overnight (16–24 h) with bicuculline 50 μM (to induce AP bursting) or 0.05% DMSO (vehicle) at 37°C. Ca^2+ ^levels at the NMDA response peak and at a time point 10 min after NMDA washout were slightly higher in spontaneously bursting and bicuculline-treated cultures when compared to the control group (control: n = 250 cells on 5 coverslips, spontaneously bursting: n = 128 cells on 2 coverslips; bicuculline: n = 316 cells on 6 coverslips, Figure 4B). This indicates that prolonged synaptic activity resulting from AP bursting slightly increases the NMDA-induced Ca^2+ ^entry and/or Ca^2+ ^release from intracellular stores, which could be due to preloading of the endoplasmic reticulum and/or a reduced sequestration and removal of Ca^2+ ^from the cytoplasm. Thus the mechanism of protection afforded by AP bursting does not involve a reduction in the Ca^2+ ^load during this toxic NMDA insult or the initial recovery period thereafter.

### Isolated extrasynaptic NMDA receptor-mediated currents and Ca^2+ ^signals are not affected by overnight AP bursting

Extrasynaptic NMDA receptors are coupled to a CREB shut-off mechanism and cell death pathways [4]. We reasoned that a specific reduction in extrasynaptic NMDA receptor numbers or function could underlie the AP bursting-induced protective effects. NMDA receptors are known to be endocytosed from extrasynaptic sites adjacent to post synaptic densities in mature dendritic spines [33,43,44]. In our cultures at the time point of recordings (11–12 DIV) spines are present (see Additional file 1). To investigate whether such changes occur after prolonged AP bursting, extrasynaptic NMDA receptor-mediated whole cell currents and Ca^2+ ^responses were measured at room temperature in single cells (see Figure 2 and text above) from overnight vehicle and AP bursting-treated neurons. All handling and medium change was performed in parallel to equalize non-specific effects. Activity of patched cells was assessed in cell-attached mode and in current clamp mode immediately after break-in (data not shown). All neurons in the bicuculline-induced AP bursting group showed regular bursts of APs in cell-attached and/or current clamp mode as well as bursting synaptic input (bursts of spontaneous EPSCs) in voltage clamp mode. 10 to 20% of cells in the vehicle-treated group showed evidence of weak but regular bursting and were discarded from the analysis.

**Figure 5:**
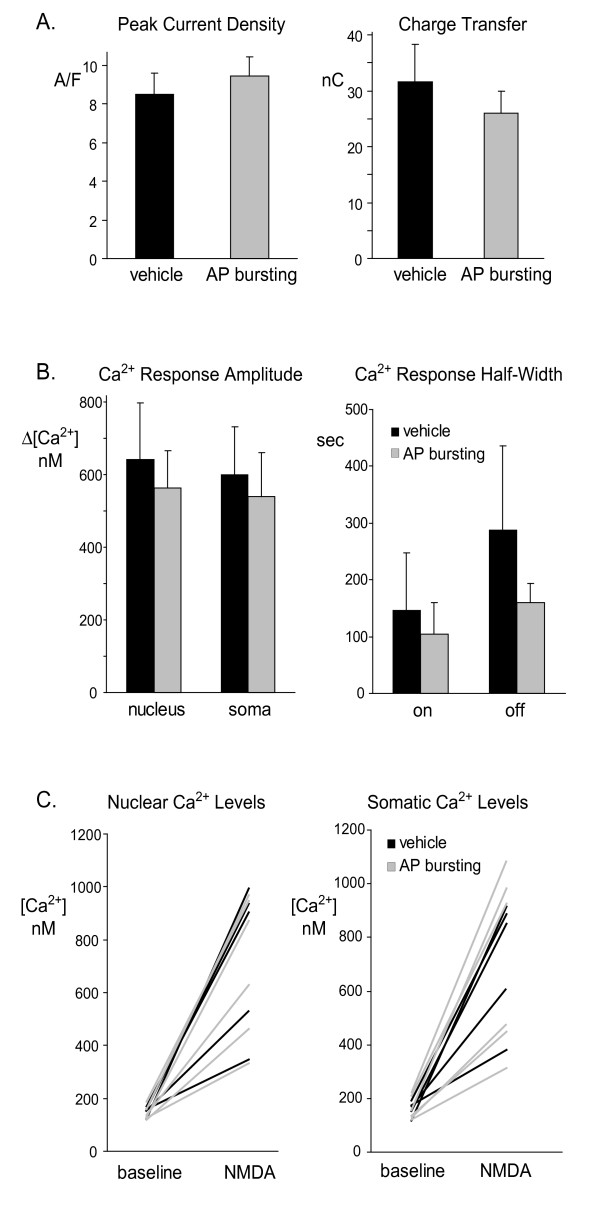
**Cumulative data indicate no significant difference in extrasynaptic NMDA receptor-mediated current or Ca^2+ ^responses between bicuculline and vehicle treated groups**. Shown are various (A) current and (B, C) Ca^2+ ^measurement parameters from responses of neurons to bath NMDA (100 μM) application following the synaptic blockade with MK-801 following overnight vehicle (black) and AP bursting (grey) treatment. All histograms show the mean and SEM. Electrophysiological measurements come from 9 vehicle and 10 AP bursting treated cells. Ca^2+ ^imaging came from 5 vehicle and 6 AP bursting treated cells. No values were significantly different between treatment groups. (A) Peak current density was measured from the maximum macroscopic current amplitude divided by the whole cell capacitance. Charge transfer was measured from the integral of the current response with respect to baseline between response onset and return to baseline. (B) Ca^2+ ^response amplitudes were measured with respect to baseline levels. Ca^2+ ^response half width was calculated for the whole cell current measured from the left (on) and right (off) half of the response waveform with respect to the time point of the response peak at a level half way between baseline and response peak. (C) Nuclear and somatic Ca^2+ ^concentrations at baseline and the NMDA response peak are shown for individual cells whereby each line connects values measured from the same cell.

A number of parameters were quantified from the current and Ca^2+ ^responses to extrasynaptic NMDA receptor stimulation (see Table 1 and Figure 5). No significant difference was seen between the AP bursting and vehicle treatment groups in any parameter including amplitude, area and half widths of either current or Ca^2+ ^response (current: n = 9 control and 10 bicuculline treated; Ca^2+^: n = 5 control and 6 bicuculline treated).

**Table 1:** Current and Ca^2+ ^responses in the vehicle and AP bursting groups were equivalent.

			vehicle	AP bursting	p value
Current	peak current	pA	607 ± 110	585 ± 82	0.83
	peak current density	pA/pS	8.49 ± 1.08	9.41 ± 1.00	0.54
	charge transfer	nC	31.67 ± 6.60	25.95 ± 4.77	0.49
	τ on	s	7.60 ± 1.14	8.21 ± 1.24	0.72
	τ off	s	19.64 ± 3.49	16.92 ± 2.76	0.54
	left half width	s	31.79 ± 3.73	39.27 ± 3.47	0.16
	right half width	s	78.72 ± 13.94	77.15 ± 10.73	0.93
Ca^2+^	peak Ca^2+^	nM	600 ± 133	540 ± 121	0.75
	Ca^2+ ^10 min later	nM	270 ± 179	149 ± 51	0.46
	Ca^2+ ^AUC	nM.s	211 ± 79	152 ± 38	0.46
	left half width	s	147 ± 101	105 ± 55	0.70
	right half width	s	287 ± 150	159 ± 34	0.34

Charge transfer and area under the curve (AUC) were calculated from the integral of the trace between the current response onset and return to baseline and between the Ca^2+ ^response onset and a point 10 min later. Current data comes from 9 vehicle and 10 AP bursting neurons of which Ca^2+ ^data was recorded in 5 vehicle and 6 AP bursting cells. All Ca^2+ ^analysis was performed from a somatic region of interest.

### Comparison of AP bursting during MK-801 exposure in EPSC and bath NMDA experiments

MK-801 is an open channel blocker requiring synchronous presynaptic glutamate release and postsynaptic depolarization to block NMDA receptors in a progressive manner over multiple synaptic activations. Although we cannot precisely quantify the number of synaptic activations in the presence of MK-801 in our recordings, we can estimate this from the number of bursts and APs occurring postsynaptically. Patch clamp and microelectrode array recordings have shown that bicuculline-induced bursts involve significant prolonged depolarization and is tightly synchronized across broad regions of the hippocampal culture [39] implying that the activity of a presynaptic and postsynaptic pair of cells is correlated and synchronized as is necessary for NMDA receptor activation. Indeed, our recordings of evoked EPSCs presented here show that synaptic NMDA receptors are rapidly blocked by our MK-801 protocol. In our recordings of bath NMDA responses we assumed that synaptic EPSCs were blocked to a similar extent to that shown in our EPSC experiments because bursting activity during MK-801 exposure was roughly equivalent to that for EPSC experiments as quantified in terms of several parameters (Table 2).

**Table 2:** Comparison of burst properties during MK-801 exposure in experiments measuring NMDA receptor-mediated EPSCs (EPSC) and those measuring whole cell current responses to bath NMDA after EPSC blockade (Extrasynaptic).

Experiment Type		EPSC	Extrasynaptic
n		5	19
incubation time	min	4.5 ± 0.7	4.9 ± 0.5
number of bursts		6.6 ± 1.4	6.2 ± 1.2
number of spikes		106 ± 34	97 ± 13
RMP	mV	-60.9 ± 4.7	-58.2 ± 1.7
Vm during bursts	mV	-50.0 ± 2.0	-53.7 ± 2.0
depolarisation	V.s	202.4 ± 73.6	187.6 ± 34.6

Average membrane potential (Vm) during bursts was calculated from the burst onset till the return to the baseline resting membrane potential (RMP). Depolarization was calculated from the integral of the membrane potential above RMP over this same period summed across all bursts for each cell.

### Large scale analysis of extrasynaptic NMDA receptor-mediated Ca^2+ ^responses

**Figure 6:**
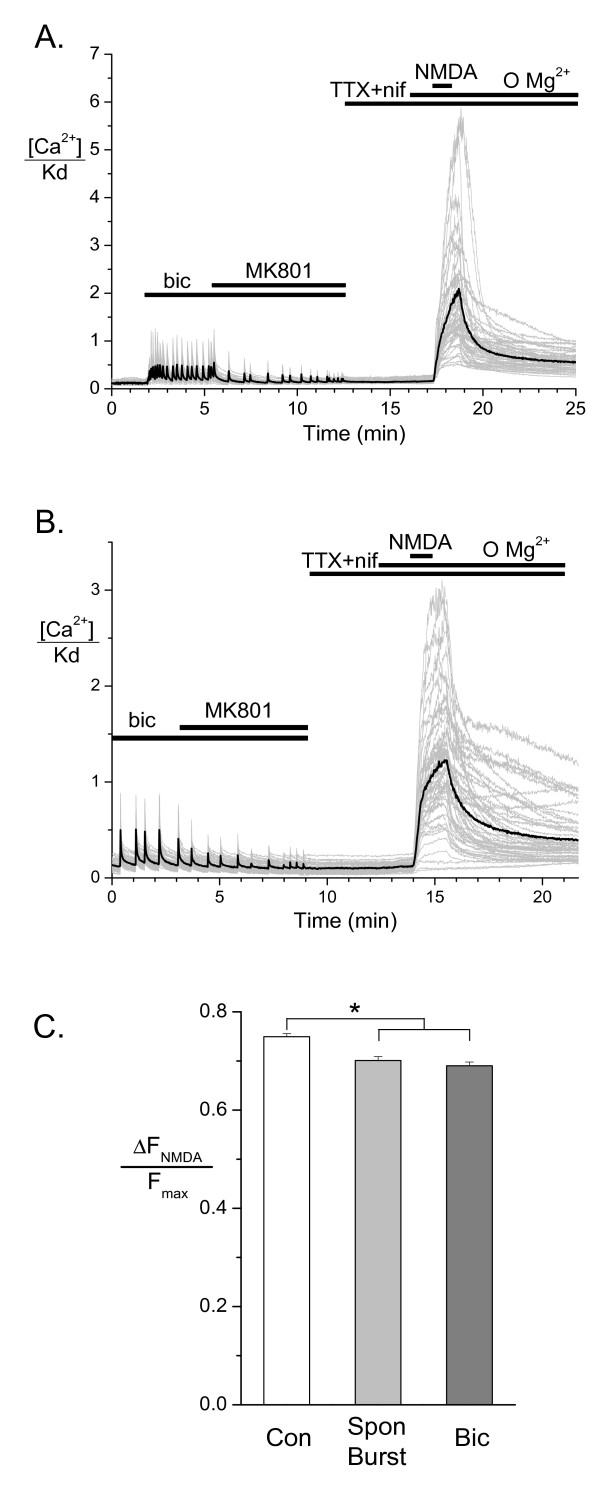
**Imaging of Ca^2+ ^responses to whole cell extrasynaptic NMDA receptor activation**. Ca^2+ ^responses were measured in cells loaded with X-Rhod 1-AM using the same protocol shown in Figure 2A except 10 μM nifedipine (nif) was included in the TTX containing solution. (A) Representative recording from a coverslip following overnight vehicle treatment. (B) Representative recording from a coverslip following overnight AP bursting. Grey traces show the results recorded simultaneously from the soma (including nucleus) of multiple cells in the same field of view and black traces show the average of these cells. (C) Cumulative data are shown from vehicle treated non bursting coverslips (Con, n = 261 cells on 5 coverslips), vehicle treated coverslips showing spontaneous bursting events (Spon Burst, n = 225 cells on 4 coverslips) and bicuculline-treated coverslips, all of which showed synchronous rhythmic AP bursting (Bic, n = 258 cells on 5 coverslips). The peak of the NMDA response is normalized to the response to ionomycin (Fmax) in the same cell. Bars represent the mean and whiskers the SEM. The vehicle treated group was significantly different from the other two groups (* p < 0.0001, ANOVA, Tukey's posthoc tests).

Having seen no difference in extrasynaptic NMDA receptor function between vehicle and AP bursting pretreatment groups under patch clamp analysis from single cells, we wished to reinvestigate this result using larger numbers of cells. For this we used a purely imaging based assay of Ca^2+ ^responses to extrasynaptic NMDA receptor activation in multiple cells (typically 50) in a single imaging field using a 20× objective. Cells were not voltage clamped. To counteract the effects of strong depolarization-induced activation of voltage-operated Ca^2+ ^channels (VOCCs) and AP-induced neurotransmitter release during NMDA exposure, we included the L-type VOCC blocker, nifedipine (10 μM), and the Na^+ ^channel blocker, tetrodotoxin (1 μM) in our solutions. As with previous experiments, MK-801 (10 μM) was applied to cells once synchronized bursting was induced with bicuculline, as seen by the regular synchronized Ca^2+ ^oscillations in all cells (Figure 6A,B). Between 8 and 12 bursts were allowed to occur in the presence of MK-801 to ensure synaptic blockade. MK-801 was then washed out in the presence of TTX and the absence of glycine before Mg^2+^-free solutions were applied followed by NMDA (100 μM) for 1 min. Due to the absence of Mg^2+^, this protocol induced much stronger Ca^2+ ^responses than 10 min of 20 μM NMDA application (see Figure 4) despite the blockade of VOCCs and APs. The responses were marginally smaller in bicuculline-treated and spontaneously bursting hippocampal neurons in comparison to the control group (control: n = 261 cells on 5 coverslips, spontaneous bursting: n = 225 cells on 4 coverslips, bicuculline: n = 258 cells on 5 coverslips, p < 0.0001, Figure 6C). This result using hundreds of cells exposed a slight difference in extrasynaptic NMDA receptor function between treatment groups which was too small to detect with our voltage clamp analysis.

## Discussion

### Validation of the bicuculline + MK-801 protocol to isolate extrasynaptic NMDA receptor function

In the presence of MK-801, NMDA receptors show a stepwise blockade in response to repeated synaptic activation. More than 90% blockade of synaptic receptors has been demonstrated in autaptic cultures after as few as 15 stimulations in 20 μM MK-801 [38] or 60 stimuli in 5 μM MK-801 [45] and similar blockade is seen in cortical brain slices after 30 stimulations in 40 μM MK-801 [46]. The rate of blockade will depend on the probability of transmitter release, the postsynaptic membrane potential, the concentrations of MK-801 and glycine and the rate of insertion of any new NMDA receptors into the synapses.

Our recordings of evoked synaptic NMDA EPSCs showed an 80% blockade after 4 min of MK-801 exposure during which 5 to 6 network bursts occurred. A similar degree of blockade after 2 min of the MK-801 protocol has been previously reported [13]. While further bursting may have produced a more complete blockade of synaptic NMDA receptors, it is necessary to limit the period of MK-801 application due to the presumed ongoing exchange of synaptic and extrasynaptic NMDA receptors through lateral movement [38]. Such exchange is likely to add blocked receptors to the extrasynaptic receptor population and may prevent complete blockade of synaptic responses due to the continuous immigration of unblocked NMDA receptors into the synapse. The use of 4-aminopyridine to enhance bursting [40] could potentially avoid this problem by accelerating synaptic blockade. Higher concentrations of MK-801 are not recommended as they sometimes block burst activity in our cultures just as NMDA receptor antagonists can shut off rhythmic bursting in slices [47] and reduce synaptically-activated spikes in the hippocampus *in vivo *[for example [48]]. Alternatively, it remains possible that the residual 20% of EPSCs not blocked by the MK-801 and bicuculline protocol represent NMDA receptor-containing synapses which were not activated by bicuculline-induced AP bursting. A similar percentage of neurons in bicuculline-treated cultures did not show bursting in cell-attached and whole cell current clamp modes. Note that residual unblocked synaptic receptors will contribute to our estimates of the extrasynaptic pool of NMDA receptors. Considering that the synaptic pool represents about 50% of the total NMDA receptor pool based on estimates from similar hippocampal cultures [13], this implies that 17% of our estimate of extrasynaptic function may be generated by synaptic receptors. Our protocol effectively isolates the extrasynaptic NMDA receptor function in standard hippocampal neuronal cultures and is a robust method to selectively activate or quantify this receptor population. This can serve as a valuable tool to study extrasynaptic NMDA receptor function *in vitro *as we have done here to explore the involvement of trafficking in the protective effects of synaptic activity.

### Overnight recurrent AP bursting only marginally alters extrasynaptic NMDA receptor function

The only significant difference found in extrasynaptic NMDA receptor function between vehicle and overnight bursting treatment groups was a slightly smaller Ca^2+ ^response in the latter. The 6% difference between these groups was highly significant but seems unlikely to account for the dramatic reduction in cell death afforded by pretreating cells with overnight bursting. However, we cannot rule out the possibility that a critical threshold for toxicity exists and that this 6% difference in extrasynaptic receptor function straddles this threshold to produce a dramatic difference in cell death between control and AP bursting groups. However, it seems more likely that mechanisms other than reduced extrasynaptic NMDA receptor function – such as Ca^2+ ^regulation of survival-promoting genes [7] – are primarily responsible for the protective effects of synaptic NMDA receptor stimulation with overnight AP bursting treatment. Our observation that extrasynaptic NMDA receptor numbers are only marginally affected by prolonged increases in synaptic activity parallel reports showing that extrasynaptic NMDA receptors in contrast to synaptic receptors are unaffected by prolonged depression of synaptic activity with ethanol [49].

Other potential mechanisms for activity-induced neuroprotection include an enhanced ability of pre-treated neurons to cope with a normally toxic Ca^2+ ^load during NMDA treatment. Regular transient Ca^2+ ^influx associated with AP bursting for an extended period might be expected to foster improved Ca^2+ ^buffering, sequestration, or extrusion mechanisms in neurons. To the contrary, however, the normally toxic 10 min NMDA application produced a slightly higher average Ca^2+ ^response in hippocampal neurons treated with overnight AP bursting (see Figure 4B). Thus prolonged AP bursting does not seem to promote neuroprotection by improving the dissipation of a toxic Ca^2+ ^load.

It is possible that the relative contribution of synaptic and extrasynaptic NMDA receptors to the Ca^2+ ^load activated by an NMDA insult determines its toxicity and that the relative contribution of these two receptor populations is altered by activity. The Ca^2+ ^response to bath applied NMDA arises from a mixed source including both synaptic and extrasynaptic NMDA receptor populations and VOCCs, which differentially couple to CREB function and mitochondrial membrane potential breakdown [4,20]. The larger Ca^2+ ^transients in cells pretreated with AP bursting (see Figure 4B) may result from an enhanced survival-promoting synaptic NMDA receptor component, which more than compensates for the observed slightly reduced extrasynaptic NMDA receptor function in this group. NMDA receptor exocytosis and synaptic function can be up-regulated by synaptic activity leading to long-term potentiation [50,51]. AP bursting in hippocampal cultures also potentiates synaptic transmission [39]. Any enhancement of synaptic NMDA receptor number or function should promote the protective effect of this receptor population.

It remains possible that overnight bursting accelerates the development of spines and synapses containing NMDA receptors thus increasing the synaptic NMDA receptor pool in a given neuron. Ca^2+ ^signaling from synaptic NMDA receptors is known to enhance dendritic outgrowth [52] and synaptic delivery of NMDA receptors occurs within hours of synaptic activation [53-55]. This scenario predicts an increase in the total (synaptic plus extrasynaptic) NMDA receptor pool consistent with the slightly higher Ca^2+ ^response to bath applied NMDA in cultures exposed to overnight AP bursting (see Figure 4B). The sprouting and/or growth of new NMDA receptor-containing synapses would also strengthen the relative contribution of synaptic receptors to the response to bath applied NMDA. This would help counteract the extrasynaptic NMDA receptor-mediated toxic effects and facilitate neuroprotection in cultures treated with overnight AP bursting. Regardless of any (minor) alteration in surface expression or distribution of NMDA receptors, the opposition of synaptic activity to NMDA receptor-mediated death converges at a level downstream of the receptor, through opposing effects on CREB function and target gene activation [2,4,7].

## Conclusion

We have developed and validated a technique for the isolation and quantitative functional assessment of the extrasynaptic NMDA receptor pool in cultured hippocampal neurons participating in neuronal networks. With this method we have shown that prolonged periods of AP bursting, which protects neurons from subsequent toxic insults, causes little change in the function of the extrasynaptic NMDA receptor pool, a receptor population linked to neuron death.

## Methods

### Hippocampal Cell Culture

Hippocampal neurons from new-born Sprague Dawley rats were prepared as described [56] except that growth media was supplemented with B27 (Gibco/BRL or Invitrogen, San Diego, CA) 3% rat serum and 1 mM glutamine. Neurons were plated onto 12 mm glass coverslips or plastic 4-well dishes at a density between 400 and 600 cells per mm^2^. All stimulations and recordings were done after a culturing period of 10 to 12 days during which hippocampal neurons develop a rich network of processes, express functional NMDA-type and AMPA/kainate-type glutamate receptors, and form synaptic contacts [3,4,57].

### The induction of network bursting and the cell death assay

Bursts of AP firing throughout the neuronal network was induced by treatment of the neurons with 50 μM bicuculline [3,39]. Bicuculline was dissolved in DMSO which did not exceed a final concentration of 0.05%. Cells with or without 16 h bicuculline pretreatment were subjected to 20 μM NMDA for 10 min at 37°C to induce cell death. After washout of NMDA cells were incubated for a further 5 h at 37°C before fixation with paraformaldehyde (4%) and stained with Hoechst 33528. Cell death was evaluated at a light microscope (Leica DM IRBE) with 40× magnification by counting condensed nuclei in 20 fields of view for every condition in each experiment. Pictures of representative areas were taken with a CCD camera (Spot Insight2; Visitron Systems, Puchheim, Germany).

### Patch clamp recordings

Whole-cell patch clamp recordings were made from cultured hippocampal neurons plated on coverslips secured with a platinum ring in a recording chamber (PM-1, Warner Instruments, Hamden, CT, USA) mounted on a fixed-stage upright microscope (BX51WI, Olympus, Hamburg, Germany). Differential interference contrast optics, infrared illumination and a CCD camera (Photometrics Coolsnap HQ, Visitron Systems, Puchheim, Germany) were used to view neurons on a computer monitor using a software interface (Metamorph, Universal Imaging Systems, Downington PA, USA).

The standard extracellular solution contained (in mM) NaCl 140, KCl 5.3, MgCl_2 _1, CaCl_2 _2, HEPES 10, glycine 0.01, glucose 30, Na-pyruvate 0.5. Patch electrodes (3–4 MΩ) were made from borosilicate glass (1.5 mm, WPI, Sarasota, FL, USA) and filled with a potassium methylsulphate based intracellular solution (containing in mM: KCH_3_SO_4_, 135; NaCl, 8; KCl, 12; HEPES, 10; K_2_-phosphocreatine, 10; Mg_2_-ATP, 4; Na_3_-GTP, 0.3; pH 7.35 with KOH). Recordings were made with a Multiclamp 700B amplifier, digitized through a Digidata 1322A A/D converter, acquired and analysed using pClamp software (Axon Instruments, Union City, CA, USA). Access (range: 10 – 28 MΩ) was monitored regularly during voltage clamp recordings and data was rejected if changes greater than 20% occurred. All membrane potentials have been corrected for the calculated junction potential of -11 mV (JPCalc program by Dr. Peter H. Barry).

### Measurement of NMDA receptor-mediated eEPSCs

Synaptic NMDA receptor-mediated currents were recorded at a holding potential of +40 mV in the presence of 10 μM glycine, 1 mM extracellular Mg^2+^, -(-)bicuculline (50 μM, Sigma), and 6-cyano-7-nitroquinoxaline-2, 3-dione (CNQX, 10 μM, Tocris). In some recordings, extracellular Ca^2+ ^concentration was reduced to 0.2 mM to reduce Ca^2+ ^mediated inactivation of the NMDA receptor [58]. Evoked excitatory post-synaptic currents (eEPSCs) were recorded in response to single 100 μs long constant current pulse stimuli (80 to 200 μA) from an A365 stimulus isolator using either a tungsten stereotrode (World Presicion Instuments, Sarasota, FL, USA) or 2 glass electrodes whose tips were positioned in contact with the tissue matrix on the surface of the coverslip, on either side of the recorded cell and at a separation of 100 to 200 μm.

### MK-801 protocol to isolate extrasynaptic NMDA receptors

Extrasynaptic NMDA receptor-mediated currents were recorded following blockade of synaptic NMDA receptors using MK-801 application during recurrent network bursting activity. 10 μM glycine was used in all our measurements of extrasynaptic NMDA receptor function to minimize glycine dependent NMDA receptor desensitization [59] and does not prime cells for NMDA receptor internalization [60]. Patched neurons were held in current clamp mode and bicuculline was applied. Once regular bursting was established (2–4 min), MK-801 (10 μM, Tocris Cookson Ltd, Bristol, UK) was added for a further 3–10 min of bursting activity. The MK-801/bicuculline solution was then washed out for 4–6 min with a solution containing no glycine and 1 μM TTX (Tocris) to halt all action potentials and NMDA receptor activation thus preventing unblocking. The cell was then voltage clamped at -71 mV and a zero Mg^2+ ^solution containing glycine (10 μM, 2 mins, 6 ml/min) was washed on for 2 min before bath application of NMDA (100 μM, 30 s, 6 ml/min). Current and calcium responses were used to quantify the total functional pool of extrasynaptic NMDA receptors for each cell. NMDA current responses typically showed an initial peak followed by decay which did not reach steady state within this application period. Ca^2+ ^responses did not reach a peak or steady state plateau within this period.

Alternatively, to test for the blockade of NMDA-receptor-mediated EPSCs following the MK-801/bicuculline protocol, bicuculline was not removed during the MK-801 washout period and CNQX (10 μM) was added instead of TTX. CNQX was just as rapid and effective as TTX in blocking all burst activity.

### Calcium Imaging

Bis-FURA2 (125 μM, Biotium, Hayward, CA, USA) was included in the recording pipette solution where indicated and allowed to perfuse the neuron intracellularly for at least 20 min before calcium measurements began. Excitation at 340 and 380 nm (bandwidth 10 and 20 nm respectively) was generated by a monochromator coupled to a light source with a 75W Xenon arc lamp (Optoscan and Optosource, Cairn, Faversham, UK). Emission was filtered (D510/80, Chroma technologies). Calibration was performed according to the recommendations of Williams and Fay [61] and Grynkiewicz [62] using the formulas:

R=Em340−Bkg340Em380−Bkg380[Ca2+]=kd(Sf2SB2)(R−Rmin⁡)(Rmax⁡−R)

where R represents the ratio of background (*Bkg*) subtracted emission (*Em*) evoked by excitation at 340 and 380 nm, *kd *represents the empirical estimate of the *kd *for bis-FURA2 using the intracellular solution (see above) with added mixtures of Ca^2+^/EGTA to give theoretical Ca^2+ ^concentrations calculated according to Portzehl *et al *[63], *R*_max _represents the ratio value achieved following perfusion with 10 μM ionomycin and 10 mM Ca^2+^, *R*_min _represents the ratio following subsequent perfusion with 10 mM EGTA in nominally Ca^2+^-free solution, *Sf*_2 _and *SB*_2 _represent the denominators used to calculate *R*_max _and *R*_min_, respectively. Regions of interest used for analysis of nuclear and non-nuclear somatic compartments were set as small regions well within and without the presumed nuclear boundary respectively. The nucleus was roughly identified by its much brighter labeling with bis-FURA2 as seen in single wavelength images. For multi-cellular Ca^2+ ^imaging experiments, cells were loaded at room temperature with membrane permeable X-Rhod1-AM (0.3 μM, dissolved in pluronic acid and DMSO, final concentration 0.1% DMSO) for 40 min followed by at least 20 min after washout to allow complete de-esterification of the dye. Overnight bicuculline treated cells were maintained in bicuculline during any loading and transfer procedure. Excitation light of 574 nm with a bandwidth of 20 nm was passed through a clean-up filter (560–580 nm, Chroma Technologies) and emission light was filtered through a 590–650 nm filter (Chroma Technologies). Somatic Ca2+ levels were quantified as:

$$\left[Ca^{2+}\right]/K_{d}=\frac{\left(F-F_{\min}\right)}{\left(F_{\max}-F\right)}$$

Where F represents the average fluorescence intensity in a somatic ROI, F_max _represents the maximal F after incubation in ionomycin (50 μM), F_min _represents the minimal F after subsequent application of EGTA (30 mM) or a saturated manganese solution (1:200). NMDA responses were normalized to F_max _(i.e. F_NMDA_/F_max_). All data are expressed as mean ± standard error of the mean.

## Authors' contributions

CPB collected all data except that of the cell death assay. CPB designed the study and drafted the manuscript. OD collected and analyzed all data in the cell death assay. HB conceived the project, assisted in its design and the drafting of the manuscript. All authors read and approved of the final manuscript.

## Supplementary Material

Additional file 1 Image of a dendrite with dendritic spines from a cultured hippocampal neuron. Confocal image (collapsed from 8 optical sections of 0.5 μm thickness) of GFP in the dendrite of a cultured hippocampal neuron at DIV 12. The cultures were prepared and treated identically to those used for the other experiments in this study except that they were transfected at DIV 8 with an expression vector for eGFP. Note the abundance of spines although many show an immature morphology. The scale bar is 10 μm. Click here for file
