# Supplementary figures and images for: A quantitative method to assess extrasynaptic NMDA receptor function in the protective effect of synaptic activity against neurotoxicity
Source: BMC Neurosci. 2008 Jan 24;9:11. doi: 10.1186/1471-2202-9-11 (PMC2267199; doi:10.1186/1471-2202-9-11)

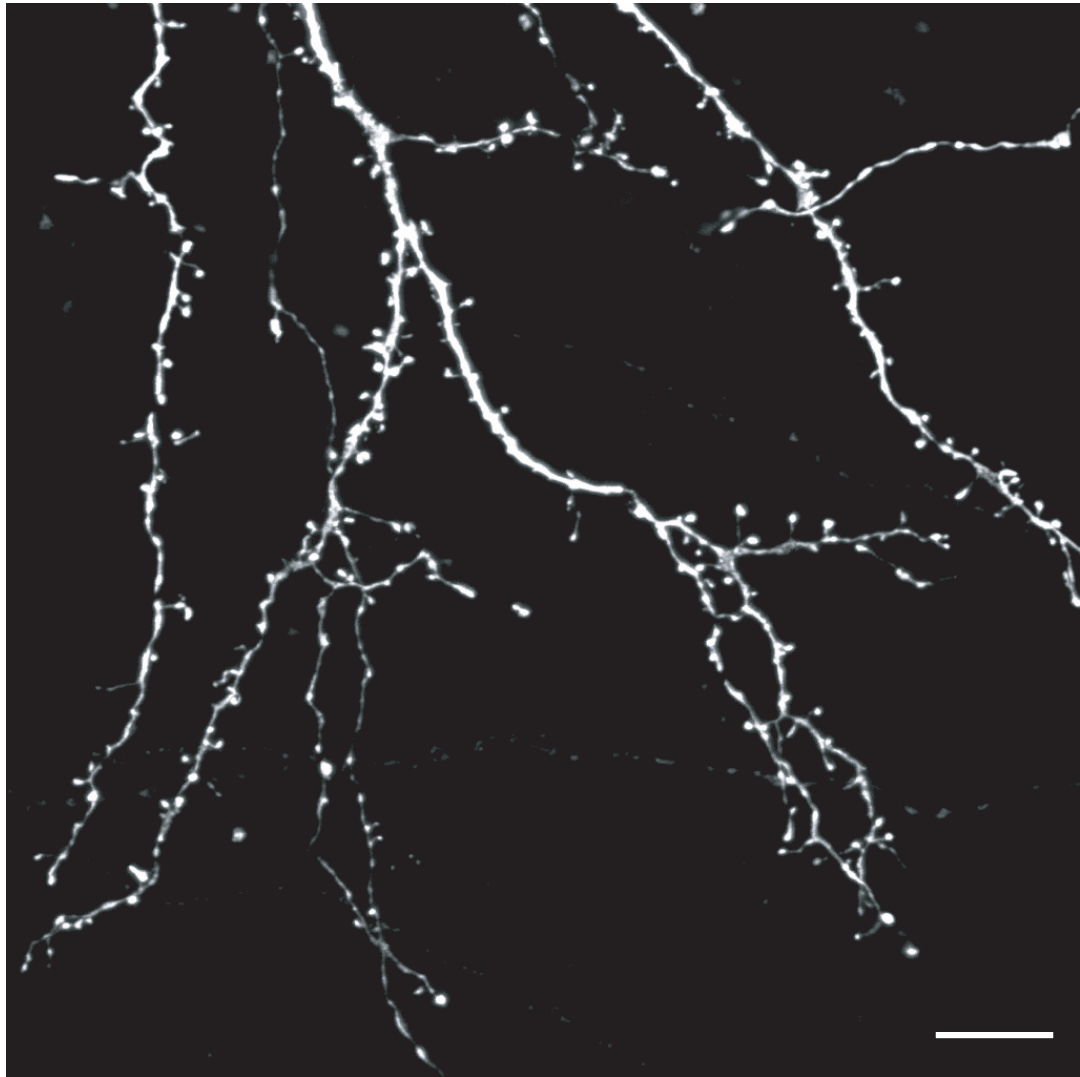

Supplement: Additional file 1 — Image of a dendrite with dendritic spines from a cultured hippocampal neuron. Confocal image (collapsed from 8 optical sections of 0.5 μm thickness) of GFP in the dendrite of a cultured hippocampal neuron at DIV 12. The cultures were prepared and treated identically to those used for the other experiments in this study except that they were transfected at DIV 8 with an expression vector for eGFP. Note the abundance of spines although many show an immature morphology. The scale bar is 10 μm. [file 1471-2202-9-11-S1.pdf]
